# Impact of Japanese Society of Pediatric Surgeons-certified supervisors and patient factors on manipulation time during single-incision laparoscopic percutaneous extraperitoneal closure: a single-center retrospective study

**DOI:** 10.1007/s00595-025-03013-1

**Published:** 2025-02-20

**Authors:** Yohei Sanmoto, Akio Kawami, Yudai Goto, Kouji Masumoto

**Affiliations:** Department of Pediatric Surgery, University of Tsukuba Hospital, 2-1-1 Amakubo, Tsukuba-shi, 305-8576 Japan

**Keywords:** Attending surgeon, Board-certified supervisor, Pediatric, Manipulation time, Single-incision laparoscopic percutaneous extraperitoneal closure

## Abstract

**Purpose:**

To assess the influence of supervisory surgeons certified by the Japanese Society of Pediatric Surgeons (JSPS) and patient-related factors on the manipulation time of single-incision laparoscopic percutaneous extraperitoneal closure (SILPEC).

**Methods:**

We retrospectively reviewed 469 SILPEC procedures that were performed between 2017 and 2023. Procedures were categorized based on whether or not the attending surgeons possessed JSPS certification as supervisors, and patient characteristics and manipulation time were compared. A multivariate analysis was also performed to evaluate the factors affecting manipulation time.

**Results:**

In male patients, procedures attended by JSPS-certified supervisory surgeon had a shorter median (IQR) manipulation time (491.5 [396, 626.3] s) than those attended by non-certified surgeons (581 [445.3, 753] s; *P* = 0.0092). However, no significant difference was observed in female patients (367 [267.8, 623] vs. 399 [269.5, 593] s; *P* = 0.94). A multivariate analysis showed that, in males, attendance by a certified supervisor was associated with shorter manipulation times, whereas a history of hernia incarceration lengthened them. Furthermore, asymptomatic contralateral patent processus vaginalis repair was associated with shorter manipulation times in females.

**Conclusion:**

Attendance by JSPS-certified supervisory surgeons significantly reduced manipulation time in male SILPEC cases. Considering patient background and procedural complexity, assigning JSPS-certified supervisory surgeons as attending surgeons may further enhance surgical efficiency.

## Introduction

Inguinal hernia repair is one of the most frequently performed procedures in pediatric surgery and typically introduced as an introductory procedure by young surgeons. Traditionally, open repair has been the standard approach; however, since Takehara et al. [1] introduced laparoscopic percutaneous extraperitoneal closure (LPEC), laparoscopic surgery has gained widespread adoption as a straightforward and reliable method for pediatric inguinal hernia repair [2–5]. Our institution has implemented single-incision LPEC (SILPEC) for inguinal hernia repair and previously reported that this procedure presents greater technical challenges in male patients, leading to longer manipulation times and significantly more peritoneal grasps with forceps than in female patients [6].

Pediatric surgeons with certification as supervisors from the Japanese Society of Pediatric Surgeons (JSPS) are distinguished by their substantial surgical experience and scholarly contributions, demonstrating their expertise in pediatric surgery. This certification ensures that they possess the necessary supervisory skills to teach young surgeons effectively. Previous studies have shown that having a JSPS-certified supervisory surgeon as the attending physician can significantly reduce the operative time for transumbilical laparoscopic-assisted appendectomy in cases of acute appendicitis [7]. However, whether or not their involvement has similar effects in other common pediatric surgeries remains unclear.

In the present study, we assessed the impact of involving a JSPS-certified supervisory surgeon as the attending surgeon on manipulation times in SILPEC for pediatric inguinal hernias. We also explored unknown factors associated with patient characteristics that may influence manipulation time.

## Methods

### Patients

The records of all patients who underwent SILPEC at the Department of Pediatric Surgery, University of Tsukuba Hospital, Tsukuba, Japan, between January 2017 and December 2023 were reviewed retrospectively, and we included only those cases in which the full surgical video was available. At our institution, SILPEC was implemented for males in February 2012 and for females in October 2008.

The following cases were excluded from the analysis: surgeries for recurrence (redo surgery), abdominoscrotal hydrocele, intraoperative catheterization, cases involving concurrent lipoma, and surgeries performed by surgeons beyond their 12th post-graduate year.

At our institution, surgeons typically perform SILPEC as operating surgeons until the 12th post-graduate year after which they transition into attending surgeons. Medical records were reviewed for patient background, including age, sex, body weight, presence of preterm birth (defined as birth before 37 weeks of gestation), presence of hydrocele, presence of ovarian sliding hernia, history of hernia incarceration, laterality of surgery including asymptomatic patent processus vaginalis (PPV), and postoperative complications including recurrence and testicular elevation. The surgical videos were also reviewed to investigate the manipulation time required for the hernia-suturing technique.

In this study, manipulation time was defined as the time from Lapa-her-closure insertion into the abdominal cavity to suture completion and until the thread was retrieved outside the body. However, each side was counted as a separate procedure in cases of bilateral inguinal hernia or when asymptomatic contralateral PPV was repaired. The surgical videos were reviewed by an experienced surgeon who performed more than 100 SILPEC procedures. In addition, we examined the post-graduate years of the operating and attending surgeons. We also assessed the attending surgeons’ experience with SILPEC as operating surgeons and whether or not they were certified as supervisors by the JSPS. This supervisory certification is awarded by JSPS to surgeons with more than 15 years of experience. Eligibility for certification requires the surgeon to have completed a minimum of 40 neonatal surgeries (including at least 1 radical operation each for esophageal atresia, intestinal atresia, diaphragmatic hernia, omphalocele, and gastrointestinal perforation) and 40 complex surgeries for non-neonatal patients (including at least 1 radical operation each for Hirschsprung’s disease, intermediate or high imperforate anus, biliary atresia, and malignant tumors). Furthermore, candidates must have delivered at least 10 conference presentations and authored 5 or more original research articles as the lead authors in pediatric surgery.

All SILPEC procedures were classified into two groups based on whether the attending surgeon was a certified supervisor from the JSPS: JSPS-certified and non-certified. Patient characteristics and surgical outcomes were compared between the two groups separately for male and female patients. For the evaluation of manipulation time, cases involving suture loosening requiring re-suturing and intraoperative peritoneal injuries requiring repair were excluded from the analysis. A multivariate linear regression analysis was also conducted to investigate patient and surgeon factors affecting manipulation time.

This retrospective study was performed in accordance with the Declaration of Helsinki (2024) and approved by the Institutional Review Board of the University of Tsukuba Hospital (approval number R06-42). An opt-out option was implemented to ensure that the participants and their guardians had the opportunity to decline the use of clinical data for research purposes.

### Surgical procedure and strategy

Detailed surgical techniques for SILPEC have been reported in our previous study [5]. At our institution, surgeons must complete a minimum of 10 SILPEC procedures in female patients as the primary surgeon before performing SILPEC in male patients. Notably, there were no other specific criteria for surgeon selection. In cases of incarcerated hernia, we generally avoid emergency surgery and instead proceed with elective surgery after successful manual reduction.

### Statistical analyses

Continuous variables are presented as medians with interquartile ranges (IQRs), whereas categorical variables are summarized as frequencies and percentages. Data between the JSPS-certified and non-certified groups were compared using chi-squared, Fisher’s exact, and Mann–Whitney U tests. Variables for the multivariate linear model were selected based on their relevance to manipulation time, considering insights from the literature, and results from preliminary analyses. Statistical significance was set at a 2-sided *P* of 0.05, and all statistical analyses were conducted using the GraphPad Prism (10.4.0) software program (GraphPad Inc., La Jolla, CA, USA).

## Results

**Fig. 1:**
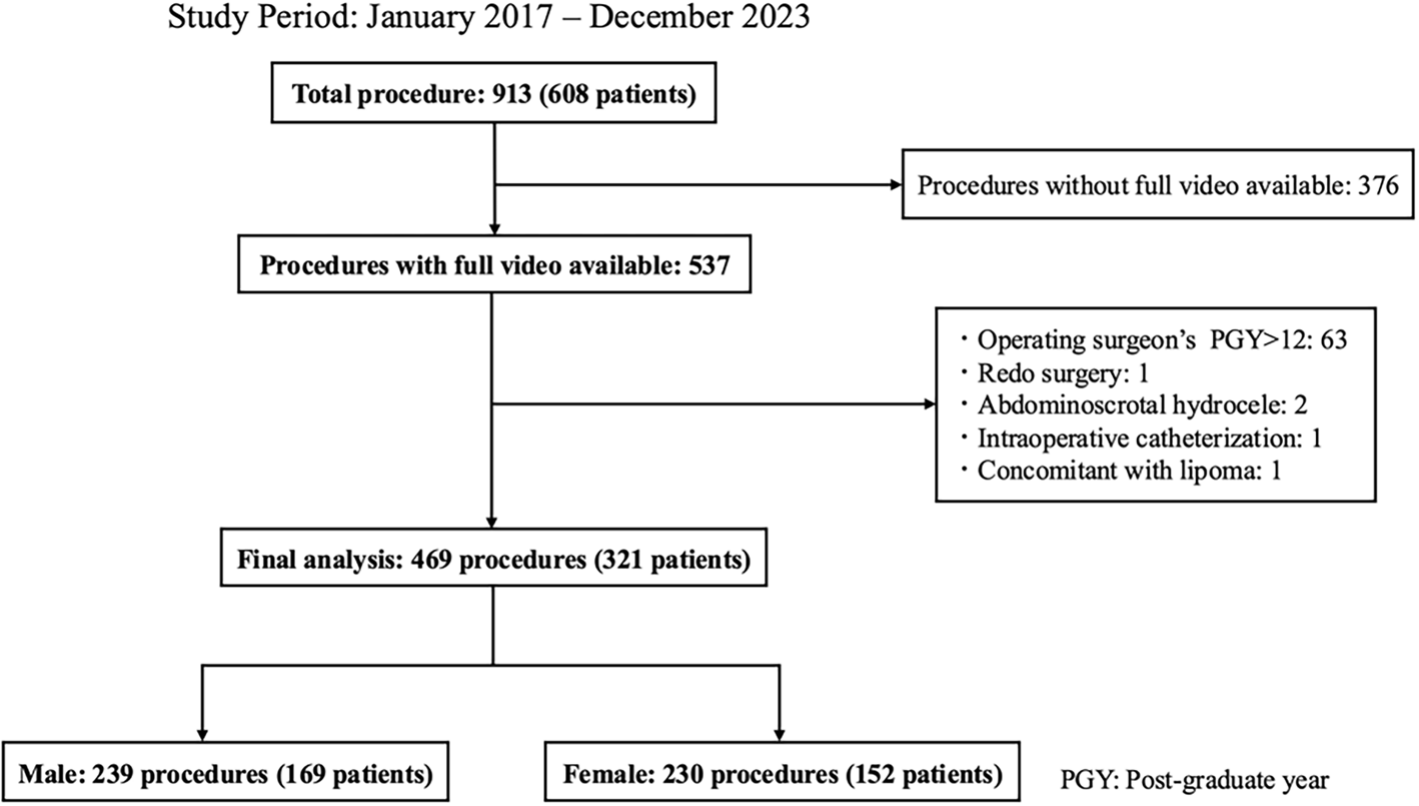
Flow chart of patient selection in the present study

During the study period, 913 procedures were performed in 608 patients. Of these, 537 procedures had complete surgical videos available for review. The exclusion criteria were as follows: procedures performed by surgeons with > 12 years of post-graduate experience (*n* = 63), redo surgery (*n* = 1), abdominoscrotal hydrocele (*n* = 2), intraoperative catheterization (*n* = 1), and concomitant lipoma (*n* = 1). A total of 469 procedures involving 321 patients were included in the analysis, comprising 239 and 230 procedures for males and females, respectively (Fig. 1). The included surgeons consisted of 18 young surgeons with a PGY ≤ 12, 12 non-certified attending surgeons, and 3 JSPS-certified attending surgeons. During the study period, one of the non-certified attending surgeons obtained JSPS certification.

**Fig. 2:**
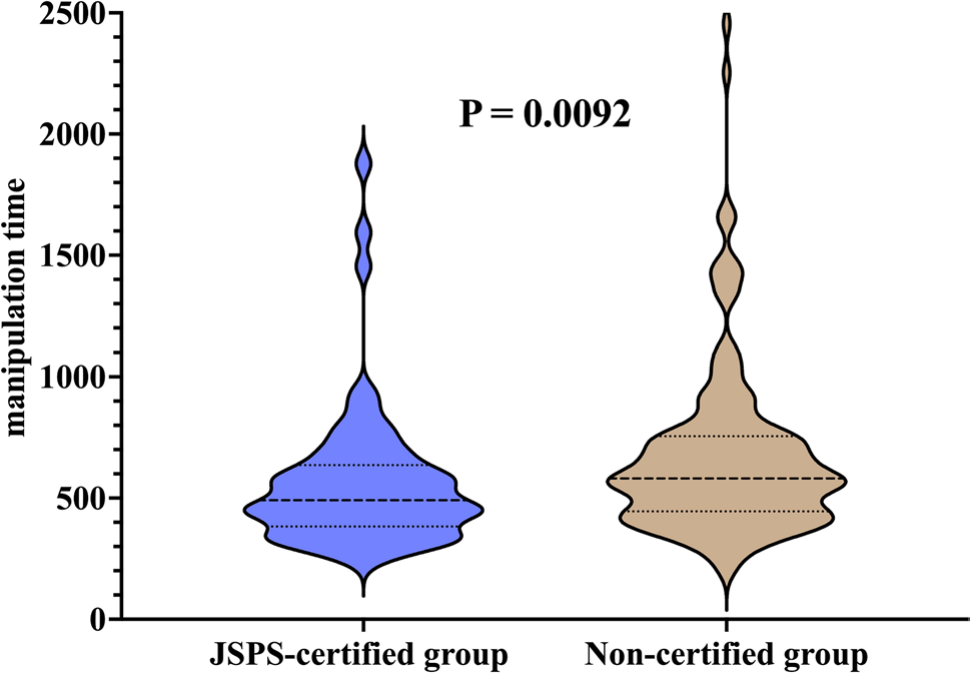
Comparison of manipulation time between the JSPS-certified group and the non-certified group in males

Notably, 51 (21.3%) of the SILPEC procedures performed in males were performed for hydrocele. The JSPS-certified supervisory surgeon served as the attending surgeon in 65 procedures (27.2%), including contributions from 2 certified supervisors. There were no significant differences between the JSPS-certified and non-certified groups in terms of patient age, weight, preterm birth, laterality of surgery, or procedures for asymptomatic PPV. A history of hernia incarceration was observed in 12 cases (5.0%): 4 and 8 cases in the JSPS-certified and non-certified groups, respectively, with no significant difference (*P* = 0.74). The median (IQR) number of days from incarceration to surgery was 17 (12.8, 29.8) days. Furthermore, the median (IQR) post-graduate years of the operating surgeon were 8 (7, 10) years in the JSPS-certified group and 7 (6, 10) years in the non-certified group (*P* = 0.11). However, the median (IQR) post-graduate years of the attending surgeon were significantly higher in the JSPS-certified group than in the non-certified group (22 [18, 25] years vs. 16 [13, 18] years; *P* < 0.001). Intraoperative complications, such as peritoneal injury and suture loosening requiring re-suturing, were observed in 11 patients; however, there was no significant difference between the two groups (*P* = 1). Postoperative recurrence occurred in one case (1.5%) in the JSPS-certified group, and postoperative testicular elevation was observed in three cases (1.7%) in the non-certified group; however, there was no significant difference between the groups (*P* = 0.27 and *P* = 0.56, respectively) (Table 1). Excluding the 11 cases with intraoperative complications, the median manipulation time (IQR) was significantly shorter in the JSPS-certified group than in the non-certified group (491.5 [396–626.3] s vs. 581 [445.3–753] s; *P* = 0.0092) (Fig. 2).

**Table 1:** Patient characteristics and operative findings in males: a comparison between groups with and without JSPS-certified supervising surgeons

	JSPS-certified group	Non-certified group	P value
	n = 65	n = 174	
Age (months), median (IQR)	40 (18, 60)	40 (15.3, 57)	0.67
Body weight (kg), median (IQR)	14.5 (10.9, 18.6)	14.1 (9.7, 17.0)	0.41
Preterm birth (< 37 weeks of gestation), *n* (%)	5 (7.7)	12 (6.9)	0.78
Left side, *n* (%)	26 (40.0)	77 (44.3)	0.66
Asymptomatic PPV, *n* (%)	14 (21.5)	46 (26.4)	0.54
History of incarceration, *n* (%)	4 (6.2)	8 (4.6)	0.74
Operating surgeon’s PGY, median (IQR)	8 (7, 10)	7 (6, 10)	0.11
Attending surgeon’s PGY, median (IQR)	22 (18, 25)	16 (13, 18)	< 0.001^***^
Attending surgeon’s past SILPEC experiences as operating surgeon, *n* (%)			
< 50 cases	37 (56.9)	38 (21.8)	< 0.001^***^
50–100 cases	0	77 (44.3)	
> 100 cases	28 (43.1)	59 (33.9)	
Intraoperative complications, *n* (%)	3 (4.6)	8 (4.6)	1
Peritoneal injury	1 (1.5)	1 (0.6)	0.47
Suture loosening requiring re-suturing	2 (3.1)	7 (4.0)	1

*IQR* interquartile range, *PPV* patent processus vaginalis, *PGY* post-graduate year, *SILPEC* single-incision laparoscopic percutaneous extraperitoneal closure, *JSPS* Japanese Society of Pediatric Surgeons

^***^P < 0.001

**Fig. 3:**
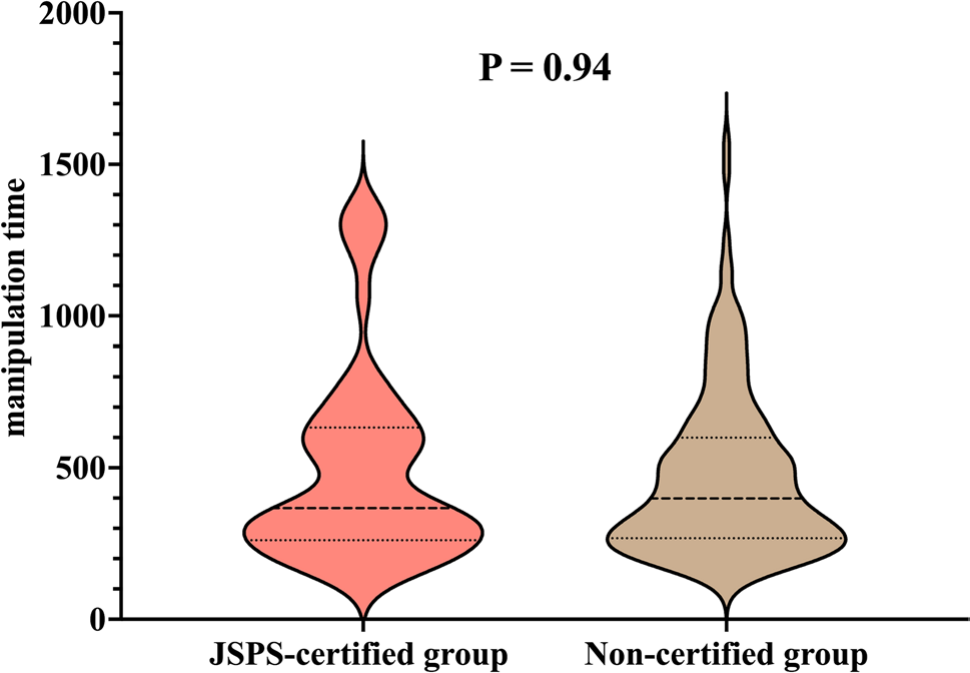
Comparison of manipulation time between the JSPS-certified group and the non-certified group in females

Furthermore, for female patients, a JSPS-certified supervisory surgeon served as the attending surgeon in 56 procedures (24.3%), which included contributions from 3 certified supervisors. However, as with the male patients, there were no significant differences in patient characteristics between the groups. In the JSPS-certified group, 1 patient (1.8%) had a history of hernia incarceration, with an interval of 89 days from incarceration to surgery. The median (IQR) post-graduate year of the operating surgeon was 6 (5, 8) years in the JSPS-certified group and 7 (6, 10) years in the non-certified group (*P* = 0.1). However, the median (IQR) post-graduate year of the attending surgeon was significantly higher in the JSPS-certified group than in the non-certified group (23 [18, 25] years vs. 15 [14, 20] years; *P* < 0.001). No intraoperative complications were observed; however, postoperative recurrence was noted in 1 case (1.8%) in the JSPS-certified group, with no significant difference between the groups (*P* = 0.24) (Table 2). The median manipulation time (IQR) was similar between the groups at 367 s (267.8, 623 s) in the JSPS-certified group vs. 399 s (269.5, 593 s) in the non-certified group (*P* = 0.94) (Fig. 3).

**Table 2:** Patient characteristics and operative findings in females: a comparison between groups with and without JSPS-certified supervising surgeons

	JSPS-certified group	Non-certified group	*P* value
	*n* = 56	*n* = 174	
Age (months), median (IQR)	63 (46, 77.5)	61.5 (43.5, 86.5)	0.73
Body weight (kg), median (IQR)	18.2 (16, 20)	17 (14.4, 22.1)	0.49
Preterm birth (< 37 weeks of gestation), *n* (%)	0	8 (4.6)	0.2
Left side, *n* (%)	25 (44.6)	84 (48.3)	0.75
Asymptomatic PPV, *n* (%)	16 (28.6)	47 (27.0)	0.96
Ovarian sliding hernia, *n* (%)	1 (1.8)	1 (0.6)	0.43
History of incarceration, *n* (%)	1 (1.8)	0	0.24
Operating surgeon’s PGY, median (IQR)	6 (5,8)	7 (6, 10)	0.1
Attending surgeon’s PGY, median (IQR)	23 (18, 25)	15 (14, 20)	< 0.001^***^
Attending surgeon’s past SILPEC experiences as operating surgeon, n (%)			
< 50 cases	39 (69.6)	14 (8.0)	< 0.001^***^
50–100 cases	0 (0)	94 (54.0)	
> 100 cases	17 (30.4)	66 (37.9)	
Intraoperative complications	0 (0)	0 (0)	1

*IQR* interquartile range, *PPV* patent processus vaginalis, *PGY* post-graduate year, *SILPEC* single-incision laparoscopic percutaneous extraperitoneal closure, *JSPS* Japanese Society of Pediatric Surgeons

^***^P < 0.001

In the multivariate regression analysis, several factors significantly affected the manipulation time for SILPEC. Among male patients, a history of incarceration (regression coefficient [B] = 292.62, 95% confidence interval [CI] 96.77–488.47; *P* = 0.0036), the operating surgeon’s post-graduate years (B = − 23.61, 95% CI − 42.85 to − 4.37; *P* = 0.016), and the involvement of an attending surgeon certified as a supervisor from the JSPS (B = − 104.03, 95% CI − 206.58 to − 1.48; *P* = 0.047) were significant predictors (Table 3). However, for female patients, factors, such as procedures for asymptomatic PPV (B = −88.01, 95% CI: − 161.52 to − 14.51; *P* = 0.019), operating surgeon’s post-graduate years (B = −39.37, 95% CI − 52.63 to − 26.10; *P* < 0.001), and attending surgeon’s post-graduate years > 18 (B = 153.94, 95% CI 81.57–226.81; *P* < 0.001), were independently influenced the manipulation time, even after adjusting for other factors (Table 4).

**Table 3:** Results of a multivariate factors analysis for factors affecting manipulation time in male patients

Independent variables	Regression coefficients	Standard error	95% CI	*P* value
(Intercept)	842.67	88.31	(668.61, 1016.73)	< 0.001
Age (months)	0.80	0.64	(− 0.46, 2.06)	0.21
Preterm birth	71.76	83.32	(− 92.46, 235.98)	0.39
Left side	− 67.16	44.24	(− 154.35, 20.03)	0.13
Asymptomatic PPV	− 72.44	51.25	(− 173.45, 28.56)	0.16
History of incarceration	292.62	99.37	(96.77, 488.47)	0.0036^**^
Operating surgeon’s PGY	− 23.61	9.76	(− 42.85, − 4.37)	0.016^*^
Attending surgeon’s PGY > 18	2.71	48.82	(− 93.51, 98.93)	0.96
Attending surgeon with a certification as a supervisor from the JSPS	− 104.03	52.03	(− 206.58, − 1.48)	0.047^*^

*CI* confidence interval, *PPV* patent processus vaginalis, *PGY* post-graduate year, *JSPS* Japanese Society of Pediatric Surgeons

*P < 0.05, **P < 0.01

**Table 4:** Results of a multivariate factors analysis for factors affecting manipulation time in female patients

Independent Variables	Regression Coefficients	Standard Error	95% CI	*P* value
(Intercept)	644.09	68.48	(509.13, 779.05)	< 0.001
Age (months)	0.79	0.54	(− 0.27, 1.85)	0.14
Preterm birth	90.90	93.92	(− 94.20, 275.99)	0.33
Asymptomatic PPV	− 88.01	37.30	(− 161.52, − 14.51)	0.019^*^
Ovarian sliding hernia	− 212.31	185.01	(− 576.93, 152.32)	0.25
History of incarceration	219.59	253.98	(− 280.96, 720.14)	0.39
Operating surgeon’s PGY	− 39.37	6.73	(− 52.63, − 26.10)	< 0.001^***^
Attending surgeon’s PGY > 18	153.94	36.72	(81.57, 226.81)	< 0.001^***^
Attending surgeon with a certification as a supervisor from the JSPS	− 60.27	42.40	(− 143.83, 23.29)	0.16

*CI* confidence interval, *PPV* patent processus vaginalis, *PGY* post-graduate year, *JSPS* Japanese Society of Pediatric Surgeons

*P < 0.05, **P < 0.01

## Discussion

This study reveals three critical new findings. First, in SILPEC procedures for male pediatric inguinal hernia, manipulation time was significantly reduced when the attending surgeon had JSPS certification as a supervisor. Second, a history of hernia incarceration in male patients undergoing SILPEC was associated with a significant increase in manipulation time. Third, in SILPEC procedures for female patients, prophylactic surgery for asymptomatic contralateral PPV was associated with a shorter manipulation time than in cases with symptomatic hernia.

Notably, studies investigating the relationship between JSPS-certified supervisory surgeons and surgical outcomes have been limited. In our previous examination of transumbilical laparoscopic-assisted appendectomy performed by young pediatric surgeons with < 10 years of post-graduate experience, we found that the absence of a JSPS-certified supervisory surgeon as attending was an independent risk factor for a prolonged operative time [7]; therefore, to our knowledge, this is the only report to date that has examined this association. In other surgical fields, a similar association has been reported, wherein procedures supervised by surgeons certified in Endoscopic Surgery Skill Qualification by the Japan Society of Endoscopic Surgery significantly reduced the operative time for laparoscopic cholecystectomy performed by young surgeons [8]. Wiseman et al. reported that in laparoscopic cholecystectomy, attending surgeons accounted for 44.5% of the variance in operative time [9]. However, resident surgeons only contributed 11.0%, underscoring the substantial impact of attending surgeon-related factors on operative time [9]. Therefore, integrating our findings with the existing literature, we can conclude that certified supervisors possess superior coaching skills compared to non-certified surgeons, enabling inexperienced surgeons to conduct procedures more efficiently. In the present study, no significant association was found between JSPS-certified supervisory surgeons and manipulation time in female patients undergoing SILPEC. This result may be due to the relative simplicity of performing SILPEC in female patients, in whom anatomical structures such as vas deferens or testicular vessels are absent, thus reducing the need for intensive guidance from the attending surgeon.

Furthermore, in male patients undergoing SILPEC, a history of hernia incarceration was significantly associated with prolonged manipulation time, likely due to inflammation induced in the peritoneum surrounding the internal ring. Miyano et al. reported that, among pediatric patients who underwent LPEC within 1 week of manual reduction of an incarcerated hernia, 61.9% exhibited edematous changes in the peritoneum surrounding the hernia ring [10]. In addition, they observed that, in early post-incarceration cases (within one week), LPEC required a significantly shorter operative time than conventional open repair; however, comparisons with non-incarcerated cases were not conducted in that study [10]. In cases of marked peritoneal edema, even experienced surgeons may encounter poorly defined tissue planes [11], necessitating particularly cautious and gentle manipulation to avoid damaging the fragile peritoneum. Furthermore, depending on the extent of edema, separating the vas deferens and testicular vessels from the peritoneum may become more challenging, potentially contributing to the extended manipulation time. In contrast, no clear association was observed between history of hernia incarceration and manipulation time in female patients undergoing SILPEC. This may be attributed to the small sample size as only one female patient had a history of incarcerated hernia in this study, which may have limited statistical power. Furthermore, the interval between manual reduction and surgery was 89 days, suggesting that any edema or inflammatory changes in the peritoneum associated with incarceration may have been resolved by the time of surgery.

In female patients, the manipulation time for prophylactic repair of an asymptomatic contralateral PPV was significantly shorter than that for symptomatic hernia repair. One contributing factor may be the smaller diameter of the opening in contralateral PPVs than on the symptomatic side. Ho et al. conducted a retrospective study of 569 pediatric patients with inguinal hernias. They found that the mean ± standard deviation diameter of the opening in contralateral PPVs, measured during laparoscopic surgery, was significantly smaller than that in symptomatic hernias (6.1 ± 2.5 mm vs. 11.2 ± 3.1 mm; *P* < 0.001) [12]. Therefore, the shorter manipulation time for asymptomatic PPV repair in our study may be due to the reduced needle passage distance required for the SILPEC procedure. In contrast, this correlation between asymptomatic PPV and reduced manipulation time was not observed in male patients, likely due to the technical complexity required to cross over the vas deferens and testicular vessels with the LPEC needle, which is a major determinant of manipulation time. Another reason for the shorter manipulation time in female asymptomatic PPV cases could be that prophylactic repair is generally performed after repair of a symptomatic hernia. In a related study, Moran-Atkin et al. reported that surgical residents who performed 10 min of warm-up simulation training within 1 h of laparoscopic surgery showed significant improvements in depth perception, bimanual dexterity, and efficiency of movements compared to a non-warm-up group [13]. At our institution, particularly in female patients, less-experienced young surgeons often perform SILPEC. Therefore, it is plausible that repeating the same technique during the initial symptomatic hernia repair might improve the efficiency, thereby reducing the manipulation time for subsequent asymptomatic PPV repair. In addition, among female patients, attending surgeons with post-graduate years > 18 years were associated with significantly prolonged manipulation times. This disparity may reflect the fact that attending surgeons with ≤ 18 post-graduate years had more opportunities to serve as operating surgeons during the early implementation phase of SILPEC than those with > 18 post-graduate years. Consequently, they were able to achieve a higher level of proficiency with the technique than their senior counterparts, which likely contributed to the observed differences in the manipulation times.

Several limitations associated with the present study warrant mention. First, this was a single-center retrospective study, which may have limited the generalizability of our findings to other institutions or broader patient populations. Second, the analysis was limited to cases with complete video records because some surgical videos were missing. The missing videos were randomly distributed; however, this limitation may have affected the comprehensive evaluation of the manipulation time by reducing the sample size and potentially introducing selection bias. Third, in the multivariate analysis of manipulation time, the clinical experience of surgeons and attending surgeons was primarily evaluated based on their post-graduate years. This approach may not fully capture the actual number of procedures performed, supervisory experience, or true level of proficiency. Finally, the relatively limited sample size in specific subgroups, such as patients with a history of hernia incarceration or cases involving ovarian sliding hernias, may have affected the statistical power of this study. Therefore, a larger sample size would be able to provide more precise insights and strengthen the reliability of conclusions regarding the factors influencing manipulation time in SILPEC.

However, despite these limitations, this study remains significant because the manipulation time for each side was evaluated individually, including in cases with contralateral PPV. In previous studies, most analyses relied on the total operative time, making it challenging to separately assess the impact of factors, such as asymptomatic PPV and hernia incarceration on manipulation time. However, in the present study, we demonstrated the effects of these individual factors.

In conclusion, our results revealed that the attending surgeon holding JSPS certification as the supervisor significantly reduced the manipulation time in SILPEC for male pediatric inguinal hernias. In addition, patient-specific factors, such as hernia incarceration in male patients and asymptomatic contralateral PPV in female patients, had a significant impact on manipulation times. These findings suggest that optimizing the selection of surgeons based on patient background and strengthening mentorship structures to support skill development among young surgeons could further enhance outcomes and improve procedural effectiveness in pediatric surgery.

## Data Availability

The data used and/or analyzed during the current study is available from the corresponding author upon reasonable request.
